# Association between body mass index and influenza antibody titer and immune cell count after influenza vaccination: an exploratory study

**DOI:** 10.1186/s12865-026-00903-y

**Published:** 2026-09-10

**Authors:** Selina Böll, Timo Schmitz, Maren Eggers, Jakob Linseisen, Christa Meisinger

**Affiliations:** 1 Epidemiology, Medical Faculty, University of Augsburg, Stenglinstraße 2, Augsburg, 86156 Germany; 2 Labor Prof. Dr. G. Enders MVZ, Stuttgart, Germany & Institut für Hygiene und Umweltmedizin, Universitätsmedizin Greifswald, Greifswald, Germany

**Keywords:** Obesity, Vaccination, Influenza, Antibody titer, Immune cell count

## Abstract

**Background:**

Obesity is associated with altered immune function and may therefore influence the response to influenza vaccination. So, the objective was to investigate whether body mass index (BMI) affected influenza-specific antibody titers and immune cell counts after vaccination.

**Methods:**

A subgroup of 25 participants from the MEGA cohort, a prospective observational study in Augsburg, Germany, aged 25–65 years, was analyzed, including 18 normal-weight individuals and 7 participants with obesity. Influenza antibody titers and immune cell phenotypes were assessed at four visits over nine months following vaccination. Differences between normal-weight and obese individuals were analyzed graphically using boxplots and tested using the Wilcoxon signed-rank test and the Mann-Whitney-U-test.

**Results:**

At baseline, no significant differences in antibody titers were observed between BMI groups. Following vaccination, antibody titers increased in both groups and remained above pre-vaccination levels throughout follow-up. In the normal-weight group, the titers for all four analyzed antibodies differed significantly at every visit compared to visit 1. This was not the case in the obese group, where only the titers for the antibodies B/Colorado/06/2017 and B/Phuket/3073/2013 differed at the different visits in comparison to visit 1. Furthermore, obese participants showed a non-significant downward trend in Treg cell proportions over time; total T-cell counts did not demonstrate clear group-specific differences. B-cell proportions showed no consistent time trend, but significant within-group differences were observed between visits 1 and 4 in the normal-weight group and visits 1 and 2 in the obese group, with significant differences between groups at visits 1 and 3.

**Conclusion:**

The findings suggest that BMI may influence cellular immune responses after influenza vaccination, whereas antibody responses appeared broadly similar among BMI groups. Given the limited sample size these observations should be interpreted as exploratory. Larger studies are needed to clarify this relationship and to inform the development of tailored vaccination strategies for obese individuals.

## Introduction

The influenza virus causes seasonal, acute respiratory illness, typically presenting with sudden onset of fever, headache, myalgia, cough, sore throat, and fatigue, lasting about seven to 14 days. Each season, there are approximately one billion influenza cases globally, resulting in 290,000 to 650,000 deaths due to severe respiratory failure, particularly in high-risk groups [1]. Of the four types of Influenza viruses (A-D), types A and B are the primary causes of seasonal epidemics. Vaccination remains the most effective method to prevent infection with these types [1].

Obese individuals are at increased risk for severe outcomes from respiratory virus infections such as influenza and COVID-19 [2, 3]. However, the impact of obesity on the immune response to influenza is not fully understood. Early studies in obese mice reported reduced vaccine efficacy, with lower antibody responses, diminished neutralizing activity, and higher viral titers [4, 5]. Sheridan et al. found that obesity was associated with a greater decline in influenza antibody titers, and decreased CD8 + T-cell activation [6]. Conversely, a recent study by King et al. did not observe significant differences in influenza vaccine effectiveness, measured by infection rates, between obese and normal weight individuals [7].

Given these conflicting findings, the present study aimed to investigate whether immune responses to influenza vaccination - including immune cell counts, phenotypes, and antibody titers - differ between normal-weight (BMI 20–24.99 kg/m^2^) and obese participants (BMI ≥ 30 kg/m^2^) in a German population-based study sample.

## Methods

### Study design and population

Participants were recruited as part of the prospective observational cohort study Metabolic health and immune status in young obese subjects - MEGA (‘Metabolische Gesundheit Augsburg’) carried out at the Institute of Epidemiology between 2018 and 2021 in Augsburg, Germany.

In the MEGA study participants from the general population aged 25 to 65 years were recruited and examined up to four times within a period of nine months. The main objective of the study was to examine immunological particularities that are associated with anthropometric measures, diet and lifestyle. Study participants were recruited in various ways, e.g. through posters and flyers, announcements in newspapers and via social media and examined in the study center of the Institute of Epidemiology. The examinations included amongst others a personal interview, self-administered questionnaires, collection of a fasting venous blood sample, anthropometric measurements and bioelectric impedance analysis. A total of 238 MEGA participants were examined at least once. The present study is based on a subgroup of study participants (*n* = 25) who received an influenza vaccine from their treating physician. The study was approved by the ethics committee of the Ludwig-Maximilian-University Munich and carried out in accordance with the Declaration of Helsinki. Written informed consent of all participants was received. The study was registered at ‘Deutsches Register Klinischer Studien’ (DRKS) with the project number DRKS00015784 (Registration Date: November 23rd, 2018; MEGA study). Clinical trial number: not applicable.

The present analysis is based on *n* = 7 participants aged 25–65 years with a BMI ≥ 30.0 kg/m² and *n* = 18 normal-weight participants (BMI 20–24.99 kg/m²) who received an influenza vaccination during the influenza season 2018/19. The antigenic composition of the influenza vaccines administered during that season was based on the WHO recommendations for the Northern Hemisphere and comprised A/Michigan/45/2015-like (H1N1)pdm09, A/Singapore/INFIMH-16-0019/2016-like (H3N2), B/Colorado/06/2017-like (B/Victoria lineage), and B/Phuket/3073/2013-like (B/Yamagata lineage) virus strains [8].

All study participants received an inactivated, quadrivalent influenza vaccine. The vaccine was manufactured using influenza viruses propagated in fertilized chicken eggs. The viruses were subsequently inactivated, and the resulting vaccine contained the relevant viral surface antigens, particularly hemagglutinin (HA).

Participants attended four visits at the study center over a 9-month period: a baseline visit (about 4 to 6 weeks before planned vaccination), 28 days after vaccination, as well as 180 days and 270 days after the baseline examination. At all study visits, participants were examined, interviewed, and bio-samples (blood, urine, stool samples) were collected. Exclusion criteria included use of antibiotics or corticosteroids within 3 weeks prior to the baseline visit, current use of immunosuppressive medication, and acute febrile illness (> 38.5 °C).

### Laboratory measurements

Blood samples collected at each study visit were preprocessed in a standardized manner immediately at the laboratory of the Institute of Epidemiology and stored at -80° Celsius. After completion of sample collection, Influenza-specific antibody titers (A/Singapore/INFIMH-16-0019/2016 (H3N2) titer, A/Michigan/45/2015 (H1N1) titer, B/Colorado/06/2017 (B/Victoria/2/87 line) titer, B/Phuket/3073/2013 (B-Yamagata /16/88 line) titer in serum were quantified at the Laboratory Enders, Stuttgart, Germany, in 2020.

### Viral samples

For the purposes of this study, a range of different influenza strains were obtained from the National Institute for Biological Standards and Control (NIBSC). The virus stocks were prepared by means of infecting MDCK cells. Aliquots of the virus stock were frozen at a temperature below − 70 °C and titrated to determine the virus dilution for neutralisation (NT).

### Cells

The Madin-Darby canine kidney (MDCK) cells were supplied by the European Collection of Cell Cultures (ECCC, Salisbury, UK). The cells were utilized at passage levels ranging from 62 to 92 and cultivated in Earle’s minimum essential medium (EMEM), with the addition of 10% fetal calf serum (FCS). For the neutralization tests, MDCK cells in EMEM, supplemented with 2% FCS and antibiotics, were seeded onto 96-well plates (20,000 cells/well, 100 µl/well) and incubated for a period of two to three hours. This and all subsequent incubations throughout the NT were carried out at 37 °C in a 5% CO2 incubator.

### Automated focus-reduction neutralisation test (AFRNT)

Neutralizing antibodies were determined as previously described by Terletskaia-Ladwig et al. [9]. Serum samples were heat-inactivated at 56 °C for 30 min and serially twofold diluted from 1:10 to 1:1280. Equal volumes of diluted serum and virus containing 10^2.3 ± 0.3 focus-forming units (FFU) were mixed and incubated for 90 min. Subsequently, 100 µl of the mixture was inoculated onto MDCK cell monolayers in 96-well plates.

After 18–24 h, cells were fixed with acetone/methanol (40:60) for 10 min, blocked with 1% BSA in PBS, and incubated with anti-influenza nucleoprotein monoclonal antibodies against influenza A (MAB8251) and B (MAB8661) at 1:10,000. Following washing, cells were incubated with anti-rabbit-HRP (1:1000), washed, and stained with AEC substrate. Plates were scanned using the EliSpot Reader (AID Diagnostika GmbH) and analyzed with AID ViruSpot software.

FRNT ND50 values were defined as the reciprocal of the highest serum dilution resulting in a ≥ 50% reduction in foci compared with the untreated virus control. Thus, for example, a serum dilution of 1:20 corresponds to an antibody titer of 20. Each serum was tested in duplicate, with replicates required to differ by no more than one twofold dilution. The lower of the two replicate titers was reported.

Antibody titers could be measured at visit 1 and 4 for *n* = 18 normal-weight and *n* = 7 obese participants. At visit 2 for *n* = 16 normal-weight and *n* = 7 obese participants and at visit 3 for *n* = 17 normal-weight and *n* = 7 obese participants.

Furthermore, at each study visit cell counts and immune phenotypes were measured via flow cytometry from fresh blood. T cells were defined as CD4 + lymphocytes and Tregs (regulatory T cells) as CD4+, CD25+, CD27- lymphocytes. B cells were characterized as CD19 + lymphocytes. Details of the gating strategy used can be found in a prior publication [10]. For the statistical analysis, percentages of the corresponding parent gate were used. Total T-cells and Tregs were measured at visit 1 for *n* = 11 normal weight and *n* = 2 obese participants; at visit 2 and 3 for *n* = 15 normal weight and *n* = 7 obese participants and at visit 4 for *n* = 18 normal weight and *n* = 7 obese people. B-cells were measured at visit 1 for *n* = 16 normal weight and *n* = 6 obese participants; at visit 2 for *n* = 16 normal weight and *n* = 7 obese persons; at visit 3 for *n* = 15 normal weight and *n* = 6 obese participants and at visit 4 for *n* = 18 normal weight and *n* = 7 obese persons.

### Statistical analysis

The population was divided into normal-weight (BMI 20–24.99 kg/m^2^) and obese (BMI ≥ 30.0 kg/m^2^) participants. Categorical variables assessed at the baseline visit were presented as absolute and relative frequencies, while continuous variables were presented as medians and interquartile ranges (IQR). The Mann-Whitney-U test was used to detect differences between the continuous variables, and the chi square test was used for differences between the categorical variables at baseline visit. The relationship between BMI and trends in influenza antibody titers and cell counts over the four visits were checked graphically using boxplots. Furthermore, in both groups, antibody titers at visits 2–4 were compared with the corresponding titers at visit 1. For this purpose, the Wilcoxon signed-rank test, a non-parametric alternative to the paired t-test, was used. In addition, differences between participants with a BMI 20–24.99 kg/m² and those with a BMI ≥ 30 kg/m² were assessed using the Mann–Whitney U test separately at each time point.

The level of significance was set to p-values < 0.05. Statistical analyses were performed using SPSS Statistics, version 29.0.0.0 (241) and R version 4.6.1.

## Results

The study included 25 participants (18 normal-weight and 7 obese). The study group included 21 female and 4 male participants. Baseline characteristics are displayed in Table 1. Median age was 44.0 years, and median BMI in the total sample was 22.5 kg/m^2^ (20.9 kg/m^2^ in the normal weight group and 36.3 kg/m^2^ in the obese group). There were no significant differences between the groups in age, smoking status, diabetes, hypothyroidism, or alcohol-consumption. However, hypertension was more common among obese participants.

**Table 1:** Demographic characteristics at baseline visit for the total sample and stratified by normal weight/obesity. Values are given as median (interquartile range; IQR) or *n* (%)

	Total *N* = 25	Normal Weight (BMI 18,5–24,9 kg/m²) *N* = 18	Obesity (BMI ≥ 30 kg/m²) *N* = 7	*P* – Values
Age (median, IQR)	44.0 (40.0–48.0)	44.0 (39.2–49.5)	44.0 (41.5–47.5)	0.916
Gender - Female	21 (84.0)	17 (94.4)	4 (57.1)	0.094
BMI (median, IQR)	22.5 (20.6–32.0)	20.9 (20.3–23.0)	36.3 (34.0–43.3)	< 0.001
Smoking				0.759
Never	16 (64.0)	12 (66.7)	4 (57.1)	
Previous	7 (28.0)	5 (27.8)	2 (28.6)	
Current	2 (8.0)	1 (5.6)	1 (14.3)	
Diabetes	1 (4.0)	1 (5.6)	0 (0.0)	1
Hypertension	5 (20.0)	1 (5.6)	4 (57.1)	0.019
Hypothyroidism	6 (24.0)	4 (22.2)	2 (28.6)	1

At baseline visit (four to six weeks prior to vaccination) influenza antibody titers were 1:20.0 for A/Singapore/INFIMH-16-0019/2016 (H3N2), 1:20.0 for A/Michigan/45/2015 (H1N1), 1:40.0 for B/Colorado/06/2017 and 1:30.0 for B/Phuket/3073/2013 in the normal weight group and 1:10.0 for A/Singapore/INFIMH-16-0019/2016 (H3N2), 1:10.0 for A/Michigan/45/2015 (H1N1), 1:20.0 for B/Colorado/06/2017 and 1:40.0 for B/Phuket/3073/2013 in the obese group, see Table 2.

**Table 2:** Antibody titers and immune cell counts over the course of four visits for normal weight and obese participants

		Visit							
		1		2		3		4	
		Median (IQR)	*P* value*	Median (IQR)	*P* value*	Median (IQR)	*P* value*	Median (IQR)	*P* value*
H3N2SGPR	BMI < 30 kg/m²	20.0 (10.0–70.0)	-	320.0 (140.0–1280.0)	**< 0.001**	240.0 (50.0–640.0)	**0.010**	160.0 (50.0–560.0)	**0.038**
	BMI ≥ 30 kg/m²	10.0 (10.0–15.0)	-	160.0 (50.0–960.0)	**0.031**	40.0 (15.0–240.0)	0.125	20.0 (15.0–200.0)	0.125
	P value**	0.205	-	0.466	-	0.302	-	0.174	-
H1N1MICH	BMI < 30 kg/m²	20.0 (10.0–80.0)	-	320.0 (140.0–1280.0)	**0.002**	160.0 (80.0–320.0)	**0.004**	160.0 (50.0–320.0)	**0.005**
	BMI ≥ 30 kg/m²	10.0 (10.0–20.0)	-	1280.0 (340.0–1280.0)	0.062	640.0 (100.0–1280.0)	0.062	640.0 (100.0–960.0)	0.062
	P value**	0.168	-	0.521	-	0.314	-	0.289	-
BCOLORAD	BMI < 30 kg/m²	40.0 (10.0–80.0)	-	240.0 (80.0–640.0)	**< 0.001**	80.0 (40.0–280.0)	**0.004**	120.0 (40.0–320.0)	**0.002**
	BMI ≥ 30 kg/m²	20.0 (10.0–40.0)	-	640.0 (200.0–960.0)	**0.031**	80.0 (80.0–240.0)	**0.031**	80.0 (80.0–120.0)	**0.031**
	P value**	0.332	-	0.323	-	0.439	-	0.980	-
BPHUKET	BMI < 30 kg/m²	30.0 (10.0–320.0)	-	640.0 (160.0–800.0)	**< 0.00** **1**	80.0 (40.0–640.0)	**0.003**	100.0 (25.0–640.0)	**< 0.001**
	BMI ≥ 30 kg/m²	40.0 (10.0–80.0)	-	640.0 (200.0–960.0)	**0.031**	320.0 (120.0–640.0)	**0.031**	160.0 (80.0–480.0)	0.062
	P value**	0.641	-	0.949	-	0.488	-	0.575	-
T cells^1^	BMI < 30 kg/m²	45.7 (39.9–54.5)	-	47.5 (39.5–56.2)	0.375	50.8 (46.5–57.0)	1	52.5 (46.0–57.4)	0.331
	BMI ≥ 30 kg/m²	55.8 (53.3–58.2)	-	45.2 (40.6–51.8)	0.500	50.1 (45.2–53.2)	1	53.5 (50.3–55.5)	1
	P value**	0.410	-	0.447	-	0.72	-	0.745	-
Tregs^a^	BMI < 30 kg/m²	6.6 (6.2–7.9)	-	6.9 (6.5–7.3)	0.695	6.9 (5.8–7.5)	0.164	6.5 (5.6–7.6)	0.240
	BMI ≥ 30 kg/m²	7.2 (7.0–7.4)	-	6.0 (5.6–8.0)	1	6.0 (5.2–6.9)	1	5.5 (4.9–6.0)	0.500
	P value**	0.769	-	0.535	-	0.315	-	**0.046**	-
B cells^b^	BMI < 30 kg/m²	8.7 (7.6–9.7)	-	8.1 (7.1–12.3)	0.632	9.6 (8.1–10.8)	0.068	9.3 (8.4–10.7)	**0.044**
	BMI ≥ 30 kg/m²	15.5 (12.2–15.9)	-	11.0 (7.6–12.8)	**0.031**	14.1 (12.9–14.4)	0.438	12.6 (10.5–13.5)	0.156
	P value**	**0.016**	-	0.671	-	**0.008**	-	0.220	-

Bold entries indicate statistically significant *P* values

^a﻿^missing data in 7 normal-weight and 5 obese individuals at baseline visit

^b﻿^missing data in 2 normal-weight and 1 obese individuals at baseline visit

^*^Wilcoxon signed-rank test

^**^Mann-Whitney-U-test

**Fig. 1:**
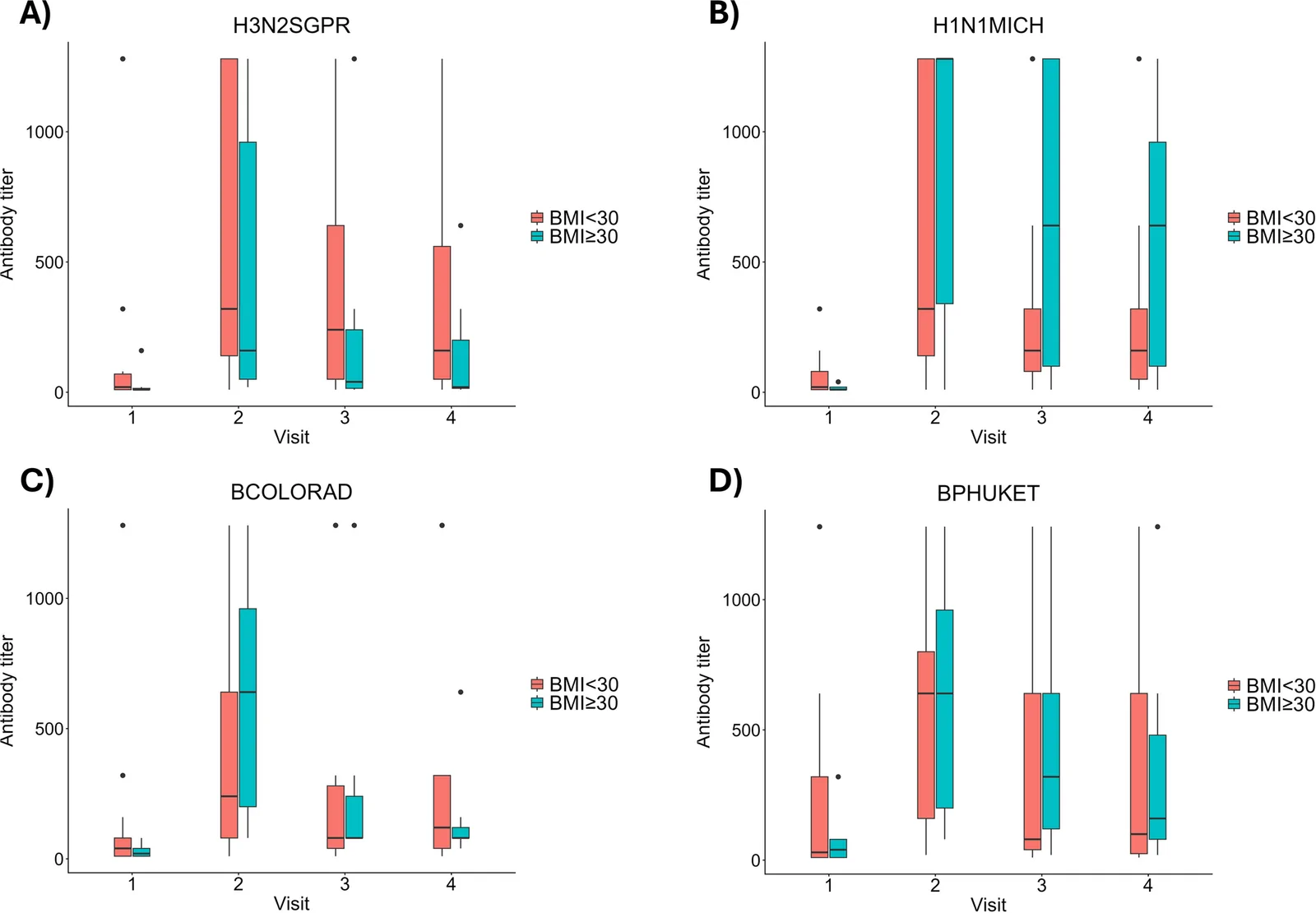
Antibody titers against (**A**) A/Singapore/INFIMH-16-0019/2016 (H3N2), **B** A/Michigan/45/2015 (H1N1), **C** B/Colorado/06/2017, **D** B/Phuket/3073/2013 over the course of the four visits between normal weight (orange bar) and obese individuals (green bar)

Figure 1 and Table 2 show the trends of the vaccination antibody titers over the four visits stratified for normal-weight and obese participants. In both groups, antibody titers increased one month after vaccination and remained above pre-vaccination levels throughout the nine-month follow-up. At all four visits, there were no significant differences between groups for any of the four analyzed antibody titers: A/Singapore/INFIMH-16-0019/2016 (H3N2) (Fig. 1A), A/Michigan/45/2015 (H1N1) (Fig. 1B), B/Colorado/06/2017 (Fig. 1C), and B/Phuket/3073/2013 (Fig. 1D). In the normal-weight group, the titers for all four analyzed antibodies differed significantly at every visit compared to visit 1. This was not the case in the obese group, where only the titers for the antibodies B/Colorado/06/2017 and B/Phuket/3073/2013 differed at the different visits in comparison to visit 1. No consistent pattern of greater increase or decrease in antibody titers was observed between obese and normal-weight participants, although A/Michigan/45/2015 (H1N1) antibody levels were higher in obese participants and remained elevated throughout follow-up compared to normal-weight participants.

**Fig. 2:**
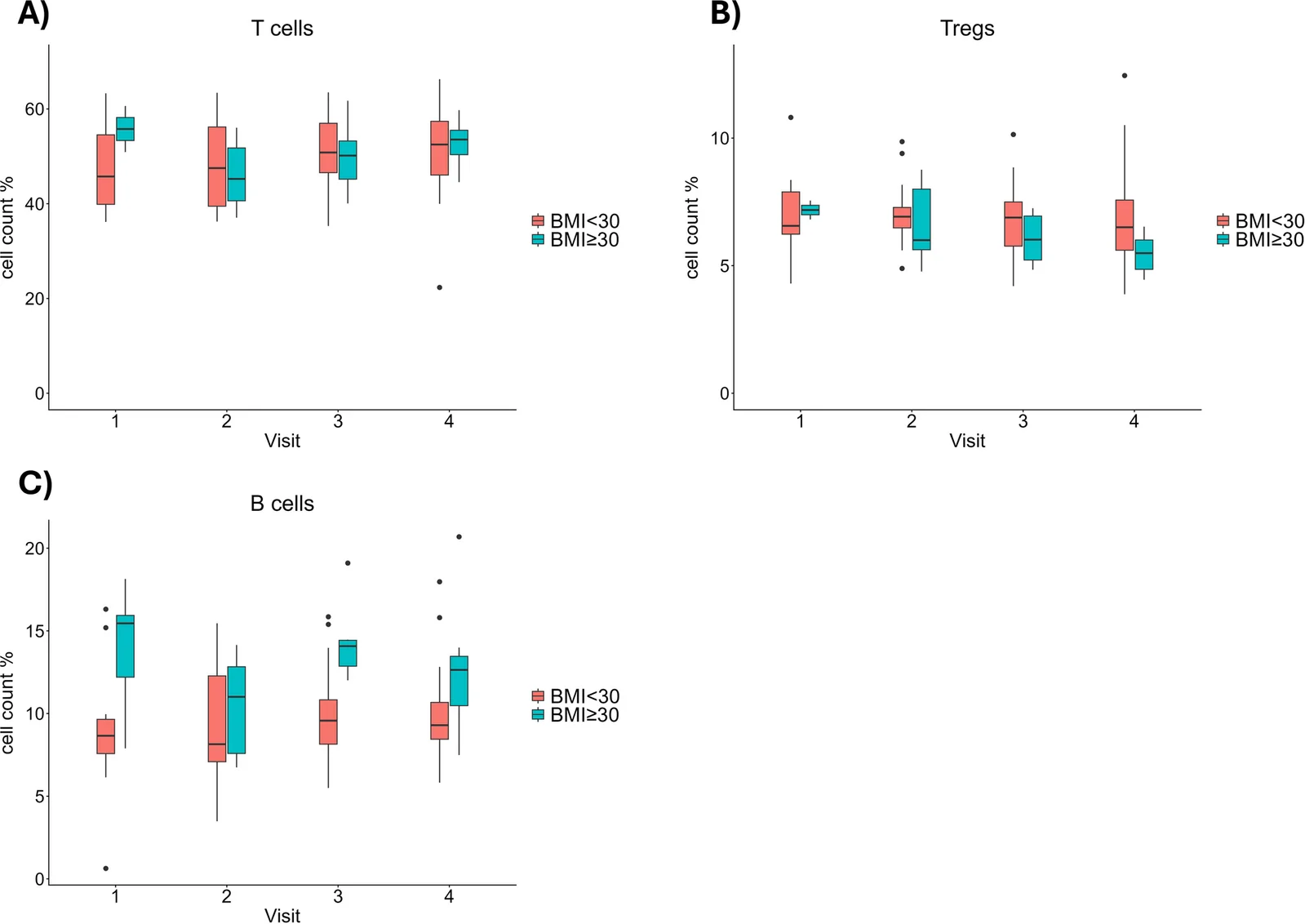
Immune cell count (**A**) Total T-cells, **B** Treg cells, **C** B-cells in percent over the course of four visits between normal weight (orange bar) and obese (green bar) participants

Figure 2 and Table 2 present changes in immune cell counts (as percentage of the corresponding parent gate) for total T-cells (Fig. 2A), Treg cells (Fig. 2B), and B-cells (Fig. 2C) over the four visits. Among obese participants, Treg cell proportions showed a decreasing non-significant trend over time whereas in normal-weight participants, these proportions remained relatively stable. However, at visit four, the Treg proportions differed significantly between obese and normal weight participants (*p* = 0.046). No clear trends nor significant differences between the groups were observed for T-cells. Although no clear trend over time was seen regarding B-cells in either group, in the normal weight group significant differences between visit 1 and visit 4 could be shown (*p* = 0.044); in the obese group, the B-cell proportions differed significantly between visit 1 and visit 2 (*p* = 0.031). Furthermore, B-cell count was significantly higher in obese compared to normal weight individuals at visit 1 and visit 3 (see Table 2).

## Discussion

The present study describes trends in immune responses (antibody titers and immune cell counts) over a nine-month period in normal-weight and obese individuals following influenza vaccination. Notably, obese participants exhibited a decreasing trend in Treg cells, and higher anti-A/Michigan/45/2015 (H1N1) antibody titers compared to normal-weight participants.

### Antibody-titer

Influenza vaccination is the most effective way for preventing severe influenza infections [1], provided the protective antibody titers are maintained throughout the influenza season. A HAI-titer (hemagglutination inhibition assay titer) ≥ 1:40 following influenza vaccination is commonly considered a correlate of seroprotection [11], although higher antibody-titers are generally associated with greater protection [12]. Previous studies have reported impaired antibody responses to vaccinations, such as hepatitis B and tetanus in obese children [13]. In the present study, antibody titers were measured before vaccination, 28 days after vaccination, as well as 180 days and 270 days after the baseline examination in obese and normal weight participants. No clear pattern of change in antibody titers over the nine-month follow-up period was observed. These findings differ from previous studies reporting accelerated declines in influenza HAI-titers in diet-induced obese mice [5] and humans [6, 14]. Furthermore, earlier research demonstrated that although obese individuals mount an antibody response to influenza vaccination, they exhibit lower IgG and IgA responses against A/H1N1 whole virus and hemagglutinin proteins compared with normal weight individuals [15].

Several biological mechanisms have been proposed to explain impaired vaccine response in obesity. Obesity is characterized by chronic low-grade systemic inflammation, including elevated levels of proinflammatory cytokines such as IL-6 and TNF-α [16]. Frasca et al. found that higher levels of TNF-α in elderly individuals were associated with reduced in-vivo antibody responses following influenza vaccination, while the addition of anti-TNF-α-antibodies enhanced serum responses in cultured B cells [17]. Another frequently proposed link between obesity and immune dysfunction is leptin, an adipocyte-derived cytokine that promotes the production of proinflammatory cytokines, including TNFα [18]. Elevated leptin concentrations have been associated with alterations in B cell maturation and function [19]. Because B-cells play a central role in the production of antibodies [20], impaired B-cell function may contribute to reduced vaccine-induced immunity and increase susceptibility to influenza infection. Despite these proposed mechanisms, the present study did not identify a consistent increase or decrease in the four investigated antibody titers. This finding may reflect the limited availability of follow-up data or may indicate that obesity had no measurable effect on antibody persistence during the nine-month observation period.

### Immune cells

Previous studies have suggested that cellular immune responses may be a more accurate predictor of protection against influenza than antibody titers alone [21]. Even in the absence of differences in seroconversion following influenza vaccination, one study reported that obese individuals had a twofold higher risk of developing influenza or influenza-like illness despite being vaccinated [22].

In addition to the production of antigen-specific antibodies by B-cells, the development of immunological memory is a key mechanism underlying vaccine-induced protection [23]. Memory T cells play an important role in mounting a faster and more effective immune response upon re-exposure to an antigen [24]. Regulatory T cells (Treg cells) are involved in the regulation of memory T cell development, and a mouse study demonstrated that Treg-cell depletion was associated with impaired formation of memory T cells [25]. Furthermore, following vaccination, B-cells differentiate into memory B cells and plasma cells, which contribute to long-term immunity through the continued production of neutralizing antibodies [26]. In the present study, a decreasing trend in Treg-cell frequencies was observed among obese participants during the nine-month follow-up period after vaccination. As Treg cells are important for the development and maintenance of immune memory, this finding may suggest possible differences in long-term vaccine-induced cellular immunity in obesity.

Several studies have reported obesity-associated alteration in T-cell function and metabolism, leading to reduced activation and impaired cytokine production [6, 27]. For example, overweight and obese individuals have been shown to exhibit altered T-cell cytokine secretion and reduced expression of CD28, a co-stimulatory molecule essential for T-cell proliferation, expansion, and survival [28]. These functional impairments may be partly explained by leptin resistance, which is commonly observed in obesity. Leptin receptors are expressed on T-cells and leptin plays an important role in T-cell differentiation, particularly toward the pro-inflammatory Th17 phenotype [29]. Dysregulation of leptin signaling may therefore contribute to chronic inflammation and impaired immune responses.

Another metabolic factor that may influence cellular immunity is insulin. Increasing evidence suggests that insulin signaling plays a critical role in T-cell proliferation, activation, and cytokine production. In a mouse model, insulin-receptor-deficient T-cells demonstrated impaired recruitment and survival of CD4 + T-cells, reduced influenza-specific CD4 + T-cells cytokine production, and diminished CD8 + T-Cell responses following influenza infection compared to wildtype mice [30]. The same study found that reduced insulin-receptor expression was associated with impaired glucose uptake and protein metabolism in activated T cells, resulting in overall functional deficits [30]. Additionally, a recent meta-analysis by Shaikh et al. discussed the potential influence of elevated insulin or insulin resistance as contributors to reduced vaccine response [31]. Because insulin resistance is frequently observed in obese individuals, impaired insulin signaling may contribute to reduced immune responsiveness and maintenance of protective memory cells. Consequently, obese individuals may be more susceptible to influenza infection despite vaccination.

### Strengths and limitation

A strength of this study is its prospective observational cohort design and the extended follow-up period. However, limitations include the small number of obese participants and missing baseline data in this group. Additionally, potential confounders such as diet, hypertension, and smoking were not fully accounted for in the present study. There was also an imbalance in group sizes (18 normal-weight vs. 7 obese participants). Obesity can result from various factors, including environmental influences such as physical inactivity and high-calorie diets, as well as genetic factors like alterations in the leptin pathway and obesity-related genes (31). Moreover, the immune cells quantified in the present study are not specific to influenza antigens. Therefore, other factors, such as concurrent infections and physiological or inflammatory conditions (e.g., stress, medications, and comorbidities) may have influenced the analyzed cell populations. Consequently, the observed changes should not be interpreted as direct evidence of an influenza antigen-specific response. Furthermore, genetic and epigenetic regulation plays a significant role in immune cell gene expression and identity, and dysregulation can lead to chronic inflammatory conditions and autoimmune diseases (32). These factors could not be controlled in the present study. Finally, the statistical power of the present study is limited due to the small sample size. Accordingly, our analyses should be considered exploratory in nature, and the findings should be interpreted primarily as hypothesis-generating and require confirmation in larger, adequately powered studies.

## Conclusion

The present study suggests a potential association between BMI and immune response to influenza vaccination, particularly regarding immune cell counts. However, due to the limited sample size, these findings should be considered exploratory. Larger studies are needed to clarify this relationship and to inform the development of tailored vaccination strategies for obese individuals, which may help address the health and economic burden of infection in this population.

## Data Availability

The datasets generated during and/or analyzed in the current study are not publicly available due to data protection aspects but are available in an anonymized form from the corresponding author on reasonable request.

## References

[1] Influenza. Updated January 24, (seasonal). https://www.who.int/news-room/fact-sheets/detail/influenza-(seasonal). 2026. Accessed January 25, 2026.

[2] Kwong JC, Campitelli MA, Rosella LC. Obesity and respiratory hospitalizations during influenza seasons in Ontario, Canada: a cohort study. Clin Infect Dis. 2011;53(5):413–21. 10.1093/cid/cir442. 21844024 PMC3156143

[3] Morgan OW, Bramley A, Fowlkes A, et al. Morbid obesity as a risk factor for hospitalization and death due to 2009 pandemic influenza A(H1N1) disease. PLoS ONE. 2010;5(3):e9694. 10.1371/journal.pone.0009694. 20300571 PMC2837749

[4] Kim Y-H, Kim J-K, Kim D-J, et al. Diet-induced obesity dramatically reduces the efficacy of a 2009 pandemic H1N1 vaccine in a mouse model. J Infect Dis. 2012;205(2):244–51. 10.1093/infdis/jir731. 22147801

[5] Park H-L, Shim S-H, Lee E-Y, et al. Obesity-induced chronic inflammation is associated with the reduced efficacy of influenza vaccine. Hum Vaccin Immunother. 2014;10(5):1181–6. 10.4161/hv.28332. 24614530 PMC4896564

[6] Sheridan PA, Paich HA, Handy J, et al. Obesity is associated with impaired immune response to influenza vaccination in humans. Int J Obes (Lond). 2012;36(8):1072–7. 10.1038/ijo.2011.208. 22024641 PMC3270113

[7] King JP, Nguyen HQ, Kiniry EL, et al. Elevated body mass index is not significantly associated with reduced influenza vaccine effectiveness. Sci Rep. 2024;14(1):21466. 10.1038/s41598-024-72081-z. 39271784 PMC11399117

[8] WHO. Recommended composition of influenza virus vaccines for use in the 2018–2019 northern hemisphere influenza season. https://www.who.int/publications/m/item/recommended-composition-of-influenza-virus-vaccines-for-use-in-the-2018-2019-northern-hemisphere-influenza-season.

[9] Terletskaia-Ladwig E, Meier S, Enders M. Improved high-throughput virus neutralisation assay for antibody estimation against pandemic and seasonal influenza strains from 2009 to 2011. J Virol Methods. 2013;189(2):341–7. 10.1016/j.jviromet.2013.03.001. 23518398

[10] Schmitz T, Freuer D, Linseisen J, Meisinger C. Associations between blood markers of glucose metabolism and characteristics of circulating lymphocytes. Clin Nutr. 2024;43(12):285–95. 10.1016/j.clnu.2024.11.004. 39546924

[11] Hobson D, Curry RL, Beare AS, Ward-Gardner A. The role of serum haemagglutination-inhibiting antibody in protection against challenge infection with influenza A2 and B viruses. J Hyg (Lond). 1972;70(4):767–77. 10.1017/s0022172400022610. 4509641 PMC2130285

[12] Hannoun C, Megas F, Piercy J. Immunogenicity and protective efficacy of influenza vaccination. Virus Res. 2004;103(1–2):133–8. 10.1016/j.virusres.2004.02.025. 15163501

[13] Painter SD, Ovsyannikova IG, Poland GA. The weight of obesity on the human immune response to vaccination. Vaccine. 2015;33(36):4422–9. 10.1016/j.vaccine.2015.06.101. 26163925 PMC4547886

[14] Frasca D, Romero M, Diaz A, Blomberg BB. Obesity accelerates age defects in B cells, and weight loss improves B cell function. Immun Ageing. 2023;20(1):35. 10.1186/s12979-023-00361-9. 37460937 PMC10351107

[15] Abd Alhadi M, Friedman LM, Karlsson EA, et al. Obesity Is Associated with an Impaired Baseline Repertoire of Anti-Influenza Virus Antibodies. Microbiol Spectr. 2023;11(3):e0001023. 10.1128/spectrum.00010-23. 37098954 PMC10269616

[16] Rodríguez-Hernández H, Simental-Mendía LE, Rodríguez-Ramírez G, Reyes-Romero MA. Obesity and inflammation: epidemiology, risk factors, and markers of inflammation. Int J Endocrinol. 2013;2013:678159. 10.1155/2013/678159. 23690772 PMC3652163

[17] Frasca D, Diaz A, Romero M, Landin AM, Blomberg BB. High TNF-α levels in resting B cells negatively correlate with their response. Exp Gerontol. 2014;54:116–22. 10.1016/j.exger.2014.01.004. 24440385 PMC3989457

[18] Procaccini C, Jirillo E, Matarese G. Leptin as an immunomodulator. Mol Aspects Med. 2012;33(1):35–45. 10.1016/j.mam.2011.10.012. 22040697

[19] Crouch M, Al-Shaer A, Shaikh SR. Hormonal Dysregulation and Unbalanced Specialized Pro-Resolving Mediator Biosynthesis Contribute toward Impaired B Cell Outcomes in Obesity. Mol Nutr Food Res. 2021;65(1):e1900924. 10.1002/mnfr.201900924. 32112513 PMC8627245

[20] Ollila J, Vihinen M. B cells. Int J Biochem Cell Biol. 2005;37(3):518–23. 10.1016/j.biocel.2004.09.007. 15618007

[21] McElhaney JE, Xie D, Hager WD, et al. T cell responses are better correlates of vaccine protection in the elderly. J Immunol. 2006;176(10):6333–9. 10.4049/jimmunol.176.10.6333. 16670345

[22] Neidich SD, Green WD, Rebeles J, et al. Increased risk of influenza among vaccinated adults who are obese. Int J Obes (Lond). 2017;41(9):1324–30. 10.1038/ijo.2017.131. 28584297 PMC5585026

[23] Sallusto F, Lanzavecchia A, Araki K, Ahmed R. From vaccines to memory and back. Immunity. 2010;33(4):451–63. 10.1016/j.immuni.2010.10.008. 21029957 PMC3760154

[24] Rogers PR, Dubey C, Swain SL. Qualitative changes accompany memory T cell generation: faster, more effective responses at lower doses of antigen. J Immunol. 2000;164(5):2338–46. 10.4049/jimmunol.164.5.2338. 10679068

[25] Bhattacharyya M, Penaloza-MacMaster P. T regulatory cells are critical for the maintenance, anamnestic expansion and protection elicited by vaccine-induced CD8 T cells. Immunology. 2017;151(3):340–8. 10.1111/imm.12734. 28295248 PMC5461106

[26] Luo W, Yin Q. B Cell Response to Vaccination. Immunol Invest. 2021;50(7):780–801. 10.1080/08820139.2021.1903033. 33779464

[27] Green WD, Rebeles J, Noah TJ, et al. Altered Metabolism and Function of T Cells Isolated from Influenza Vaccinated Obese Adults. FASEB J. 2017;31(S1). 10.1096/fasebj.31.1_supplement.434.3.

[28] Paich HA, Sheridan PA, Handy J, et al. Overweight and obese adult humans have a defective cellular immune response to pandemic H1N1 influenza A virus. Obes (Silver Spring). 2013;21(11):2377–86. 10.1002/oby.20383. PMC3695020 23512822

[29] Shirakawa K, Sano M. Drastic transformation of visceral adipose tissue and peripheral CD4 T cells in obesity. Front Immunol. 2022;13:1044737. 10.3389/fimmu.2022.1044737. 36685567 PMC9846168

[30] Tsai S, Clemente-Casares X, Zhou AC, et al. Insulin Receptor-Mediated Stimulation Boosts T Cell Immunity during Inflammation and Infection. Cell Metab. 2018;28(6):922–e9344. 10.1016/j.cmet.2018.08.003. 30174303

[31] Shaikh SR, MacIver NJ, Beck MA. Obesity Dysregulates the Immune Response to Influenza Infection and Vaccination Through Metabolic and Inflammatory Mechanisms. Annu Rev Nutr. 2022;42:67–89. 10.1146/annurev-nutr-062320-115937. 35995048 PMC10880552

